# Influence of seasonal and geographic variation on the anti-HSV-1 properties and chlorogenic acids content of *Helichrysum aureonitens* Sch. Bip

**DOI:** 10.3389/fmolb.2022.961859

**Published:** 2022-08-25

**Authors:** Wilson Bamise Adeosun, Garland K. More, Paul Steenkamp, Gerhard Prinsloo

**Affiliations:** ^1^ Department of Agriculture and Animal Health, University of South Africa, Johannesburg, South Africa; ^2^ College of Agriculture and Environmental Sciences, University of South Africa, Johannesburg, South Africa; ^3^ Research Centre for Plant Metabolomics, Department of Biochemistry, University of Johannesburg, Johannesburg, South Africa

**Keywords:** seasonal variation, antiviral, *Helichrysum aureonitens*, herpes simplex virus type 1 (HSV1), chlorogenic acids, UPLC-QTOF-MS, medicinal plants

## Abstract

Pharmacological studies conducted in the past revealed the potential source of medicinal plants in the development of novel medicines. The phenolic contents of medicinal plants containing chlorogenic acids (CGA) have been linked to a variety of therapeutic effects, especially antiviral activity. *Helichrysum aureonitens* is a medicinal plant which has been reported to contain chlorogenic acids compounds and has also shown antiviral activities against a number of virus species including Herpes Simplex Virus-1 (HSV-1). In this study, the aim was to determine both the influence of seasonal variation and locality on the antiviral properties of *H. aureonitens*. Since chlorogenic acids have been reported as potent antiviral compounds, these compounds were targeted to determine the effects of locality and seasonal change on the chlorogenic acid profile, and subsequent antiviral activity. The ultra-performance liquid chromatography-quadrupole time-of-flight mass spectroscopy (UPLC-qTOF-MS) was employed to determine the metabolic profile variations of three derivatives of chlorogenic acids-caffeoylquinic acid (CQA), dicaffeoylquinic acid (DCQA) and tricaffeoylquinic acid (TCQA) in the harvested plants growing in two diverse geographical climates and two different seasons (spring and autumn). Using the cytopathic effect (CPE) reduction approach, twenty-six samples of the plants’ leaves and stems collected during spring and autumn at Telperion nature reserve in Mpumalanga and Wakefield farm, Midlands in KwaZulu-Natal region of South Africa were evaluated for anti-HSV activity. The MTT assay was used for the cytotoxicity evaluation of the extracts prior to antiviral determination. Seventeen (mostly spring collections) of the twenty-six extracts examined were found to have considerable anti-HSV activity as measured by a reduction in tissue culture infectious dose (TCID_50_) of less than 10^5^. The UPLC-qTOF-MS result revealed that dicaffeoylquinic acid (DCQA) is the most abundant, with higher concentrations in both regions and seasons. 3-CQA was also shown to be the most abundant isomer of caffeoylquinic acid in this investigation.

## 1 Introduction

*Helichrysum aureonitens* whose common name is golden everlasting is a tufted perennial herb widely distributed across South Africa. As an important medicinal plant, it is used for the treatment of many infections including enuresis, menstrual pain and influenza (Hutchings and van Staden, 1994; Meyer and Afolayan, 1995). The phenolic compound galangin extracted from *H. aureonitens* showed significant antiviral activity against HSV-1 (Meyer et al., 1996; Meyer et al., 1997). The plant has also been reported to contain various chlorogenic acids, well known for the antiviral activity, especially against HSV.

Humans play host to HSV, a big DNA virus belonging to the family *Herpesviridae*. The virus exists in two categories: HSV-1 known as oral herpes and HSV-2 otherwise referred to as genital herpes. The WHO reported that close to 67% of the world’s population of below 50 years are infected with HSV-1 (World Health Organisation, 2016). It is a highly communicable disease that is endemic worldwide. It can lead to a variety of infections ranging from moderate to severe, including cold sores, corneal blindness, encephalitis and keratitis, especially in immunocompromised persons. HSV infections increase the likelihood of contracting HIV infection and thus contributing to HIV epidemy (Jiang et al., 2016). The disease development begins with severe infection of the mucosal tissue and progresses to sensory neurons, where it creates a latent infection (Cliffe et al., 2013).

Orthodox antiviral medicines such as interferon and ribavirin available for treatment of viral related infections are only potent against most viruses *in vitro* while they are often ineffective when administered to patients (Ben-Shabat et al., 2020). Furthermore, many of the medications lack specificity when it comes to treatment of particular viral infection (Jiang et al., 2015). Three classes of drugs were approved for the treatment of HSV infections which are: acyclic guanosine analogues, acyclic nucleotide analogues and pyrophosphate analogues. They function primarily through the targeting of viral DNA replication (Jiang et al., 2016). There is however a high chance of drug resistance in cases of long-term treatment (Castelo-Soccio et al., 2010; Kakiuchi et al., 2013). A growing concern on antiviral drug resistance especially in immunocompromised patients due to prolonged exposure to antiviral drug treatments leading to development of resistant strains have been reported (Strasfeld and Chou, 2010; Pennings, 2013; Feder et al., 2021). This necessitates a need to look into the present therapy methods (particularly resistant virus strains therapies) with a view to enhance and augment them with the discovery of novel antiviral agents from plant sources for the treatment of resistant viral infections.

Various medicinal plants have been reported to have potent antiviral effects at various stages of viral development (Serkedjieva et al., 1990; Abd-Elazem et al., 2002; Karimi et al., 2016). Recent studies have also reported antiherpetic derived chemicals from natural sources, especially phenolic compounds including flavonoids and curcumin for the safe treatment of HSV infection (Wianowska and Gil, 2019; Treml et al., 2020; Šudomová et al., 2022). Many studies have shown that plant species of the genus *Helichrysum* are known to possess antiviral activities (Sindambiwe et al., 1999; Dhakad et al., 2017; Kutluk et al., 2018). For example, Meyer and Dilika (1996) in their study on the evaluation of antiviral potentials of *H. aureonitens* have established remarkable activity of the plant on HSV-type 1 as evidenced by the absence of a cytopathic effect on human lung fibroblasts. The activity of *Helichrysum* species has often been linked to the presence of phenolic compounds and specifically the abundance of chlorogenic acids.

Chlorogenic acids (CGA) have been reported to have antioxidant, anti-inflammatory, anti-HIV, anti-HBV, anti-influenza A (H1N1/H3N2), anti- Enterovirus 71 (EV71), anti- porcine reproductive and respiratory syndrome virus (PRRSV), anti-diabetes, anti-HSV and carcinogenic properties and as such are regarded as greatly beneficial to the health of humans (Neyts et al., 1992; Hemmerle et al., 1997; Bourne et al., 1999; Kwon et al., 2000; Kweon et al., 2001; Khan et al., 2005; Wang et al., 2009; Ikeda et al., 2011; Cheng et al., 2013; Li et al., 2013; Ding et al., 2017; Prinsloo and Vervoort, 2018). As common secondary metabolites in plants, CGA are found in abundance in coffee, tea, potatoes, and a variety of other vegetables and fruits. The main source of CGAs in the human diet is coffee beans and commercial coffee products (Clifford et al., 2003).

The chemical profile of medicinal plants determines their biological activities (Soni et al., 2015). Active metabolites in plants are the outcome of long-term interactions between plants and their environment, and their production and modifications have a significant relationship and association with the environment (Blanch et al., 2007). Some compounds can only be synthesized under certain conditions, or there can be an upsurge in the levels of some compounds in specific environmental conditions (Filella et al., 2007).

Generally, variation in seasons among other factors is an important contributor to both the quality and quantity of active compounds responsible for the biological activities of plants (Mahajan and Tuteja, 2005; Gouveia and Castilho, 2012). Results showed that variations in season influence the distribution of biochemical compounds in plants leading to differing biological activities. Furthermore, individual plants of the same species growing in different sites at the same location may possess different biological activities in what seems to be influenced by a microclimate with a local set of atmospheric conditions that are particular to different sites.

Liquid chromatography- mass spectrometry (LC-MS) analysis has become a standard approach for exploring the quantity, quality, and chemical variety of plant metabolites as a result of recent technological and methodological breakthroughs in both liquid chromatography (LC) and mass spectrometry (MS). The goal of targeted LC-MS metabolite analysis is to detect and quantify the target metabolites of interest (Shimizu et al., 2018). Although CGA has been reported in a number of *Helichrysum* species including *H. aureonitens* (Albayrak et al., 2010; Heyman et al., 2015; Yazdi et al., 2019; Vujić et al., 2020), no study has been conducted to determine the specific derivatives of CGA present in *H. aureonitens* leaves in response to seasonal variations and different geographical locations.

In this study, the influence of seasonal variation and different sites in the same locality on the antiviral properties and chemical profile of *H. aureonitens* leaves using CPE reduction approach and UPLC-qTOF-MS analysis respectively were investigated. The results showed that dicaffeoylquinic acids were the most abundant derivative of chlorogenic acids during both seasons and in all the collection locations.

## 2 Materials and methods

### 2.1 Plant material collection

Two batches of *H. aureonitens* whole plant materials were obtained at two climatically different regions in two different seasons of the year namely spring (late October 2017) and autumn (early May 2018). The regions are Telperion nature reserve, located in Mpumalanga (25.7039°S, 28.9814°E) and Wakefield farm, in the KwaZulu-Natal Midland region (29°30′0″S and 29°54′0″). Three to four batches of plant samples were obtained from each site in both regions and were transferred from the sites of collection into large brown paper bags and carefully transported until reaching the laboratory. Plant materials were identified and representative voucher specimens with the names WAHA-01, WAHA-02, WAHA-03, and WAHA-04 were deposited in the UNISA Science Campus horticulture centre’s herbarium. Plants were allowed to air dry in the laboratory at ambient temperature and in natural light until they were completely dry. Additionally, each plant’s stems and leaves were removed and placed in clear cellophane bags for further investigation.

### 2.2 Preparation of plant extracts

Plant extraction was carried out as reported by Eloff (1998). A 10:1 ratio of acetone to plant materials (50 g of plant material to 500 m of acetone) was introduced into the centrifuge tube (1998). Samples were shaken overnight at 130 rpm in a shaker (Thermo Fisher Scientific, United States), then centrifuged for 10 min at 3,000 rpm in an Eppendorf microcentrifuge (5427R, Germany), with the supernatant transferred to a glass vial. The residual solvent was removed from the extracts that had already been deposited in pre-weighed glass tubes and stored at room temperature using a stream of room temperature air.

### 2.3 Rainfall and temperature data

The South African weather services provided both rainfall and temperature data. Cedara is the closest station to Wakefield farms, a place roughly 15 km apart with meteorological characteristics that are considered to be similar to Wakefield farm. Witbank is the nearest station to Telperion with similar weather data. Table 1 displays the average minimum and maximum temperatures as well as average daily rainfall statistics for the two locations from August 2017 to June 2018.

**TABLE 1:** Average minimum and maximum temperatures and monthly rainfall data between August 2017 and June 2018 for Cedara and Witbank.

Month	Wakefield (Cedara data)			Telperion (Witbank data)		
	Avg daily	Avg daily	Total monthly	Avg daily	Avg daily	Total monthly
	Max T ^°^C	Min T ^°^C	Rainfall (mm)	Max T ^°^C	Min T ^°^C	Rainfall (mm)
August	20.8	5.2	3.6	21.3	5.6	3.8
September	24.5	8.9	9.6	26.8	9.8	27.2
October	22.3	9	110	24.5	10.7	83.8
November	23.7	10.7	105.8	27	11.9	109.2
December	23.8	12.6	79	26.5	14.1	153.2
January	27.3	14.2	79.4	27.9	13.9	71.8
February	26.5	15.3	170.2	26.3	15	75.8
March	25.7	13.7	124.2	26.0	13.4	148.4
April	23.9	12.2	52.4	23.9	12.0	26.0
May	21	6.7	31.8	21.4	7.0	24.6
June	20.2	3.4	1	20.1	4.5	0.2

### 2.4 Preparation of assays

#### 2.4.1 Virus culture and assay

Herpes simplex virus type 1 (HSV-1; 15,577 strain) purchased from Anatech analytical technology (South Africa) was used in the study. The strain is susceptible to the standard drug acyclovir (Garber et al., 2021). Vero cells (African green monkey kidney) (Cellonex Separation Scientific, Roodepoort, South Africa) were used to grow the virus while Minimal Essential Medium (MEM) supplemented with 5% (v/v) fetal bovine serum (FBS), 2 mM L-glutamine, non-essential amino acids (×1) and 100 g/ml streptomycin [Celtic Molecular Diagnostics SA (Pty) Ltd., Cape Town, South Africa] was used as a medium to propagate the Vero cells and then incubated at 37°C in an atmosphere of 5% CO_2_ and observed daily for evidence of cytopathic effect. After that, the flasks were frozen at −70°C and then thawed to release cell-associated virus. The Reed and Muench method was used to estimate the TCID_50_ values of each of the viruses employed in this investigation (Reed and Muench, 1938).

#### 2.4.2 Cytotoxicity assay

The 3-(4,5-dimethylthiazol-2-yl)-2,5-diphenyltetrazolium bromide colorimetric (MTT) assay as described by Mosmann (1983) was used to measure cell viability. The process for determining the cytotoxic concentration of the plant extracts began with seeding the Vero cells cultured in Dulbecco’s modified eagle’s medium (DMEM; Gibco) at a density of 25,000 cells/well of 96-well flat bottom cell culture microtiter plates and incubated for 24 h to allow the cells to attach to the 96 well plates. After 24 h of incubation at 37°C in a humidified 5% CO_2_ environment, cells were exposed to various doses of the extracts dissolved in 10% Dimethyl sulfoxide (DMSO) (Sigma-Aldrich^®^ Darmstadt, German) and further incubated for 24 h. The media was discarded and replaced with fresh 20 µl of MTT prepared in phosphate buffered saline (PBS) Gibco, which was added and the plates were incubated for 4 h followed by the addition of 100 µl (DMSO) which was pipetted into each well. The plates were carefully rocked to disintegrate formazan crystals which are the by-products of the tetrazolium salt. The presence or absence of purple formazan colour as observed in the wells gives an indication of the cytotoxic effect of the extracts on cells. The optical density (OD) of the MTT was read at a wavelength of 570 nm and at a reference wavelength of 630 nm using an ELISA microplate reader (VarioSkan Flash, Thermo Fisher Scientific, Vantaa, Finland). Cytotoxicity results were expressed as extract concentration that are lethal to cell growth by 50% (LC_50_), calculated using a linear regression equation. Vero cells monolayers treated with Acyclovir (Sigma-Aldrich^®^ Darmstadt, German) were used as positive control, while untreated cells were used as negative control. Cytotoxicity experiments were done in triplicates.

#### 2.4.3 Antiviral assay

A method by Barnard et al. (1992) was used with some modifications to determine the antiviral activity of the extracts. Extract concentrations that were not toxic to the cells were diluted in DMEM containing 5% FBS and 1% penicillin/streptomycin (PenStrep, Sigma-Aldrich^®^ Darmstadt, German), to which equal volume of HSV (20 µl) at an infective titre of 10^2^ TCID_50_/ml was added. The combined extract-virus solution was incubated at 37°C for a time range of between 1 and 3 h. Cell monolayers grown in 96-well plates that were confluent had their growth media removed. One-hundred (100) µl of the mixture of extract-virus was added to the cells at each (10 μg/ml) concentration and incubated for a time period that ranged between 1 and 5 days depending on when cytopathic effect (CPE) was observed. Untreated infected cells were used as negative control and Acyclovir treated infected cells were used as positive control. Furthermore, serially diluted solvent control (10% DMSO) was included in experiments. The presence of CPE was confirmed by microscopic examination. Plant extracts that limit viral growth at dilution range above 10^5^ indicate mild to weak activity, whereas those that reduce viral infectivity at dilutions ranging between 10^5^ and 10^0^ have strong activity. Experiments were conducted in triplicates and two independent experiments were conducted.

### 2.5 Chemical profile determination

#### 2.5.1 Method

An established method was used, proven to separate the different chlorogenic acids in plant extracts (Clifford et al., 2003). Reference standards for CQA, DCQA, and TCQA of these compounds were used for identification. Dried leaves of *H. aureonitens* (50 mg) were pulverized and extracted with 1.5 ml of 80% methanol (LC-grade and ultrapure LC-grade water). Extracts were later homogenized and sonicated in an ultrasonic bath for 5 min followed by centrifugation of the homogenates for 15 min at 15,000 rpm. Each sample was filtered through a 0.22-micron nylon syringe filter (Sartorius Minisart RC 4), and the filtrate concentrated by evaporation to dryness. The dried extract was reconstituted in 300 µl of 50% methanol and pipetted into HPLC glass vials (2 ml). Before analysis, aliquots of extracts were produced in triplicates and kept at −20°C.

#### 2.5.2 Ultra-performance liquid chromatography analysis

A Waters Classic UPLC, coupled in series to a Waters SYNAPT G1 HDMS mass spectrometer was used to generate full scan accurate mass data. Optimization of the chromatographic separation was done utilizing a Waters HSS T3 C18 column (150 mm × 2.1 mm, 1.8 µm) and the column temperature controlled at 60°C. A binary solvent mixture was used consisting of water (Eluent A) containing 10 mM formic acid (natural pH of 2.4) and acetonitrile (Eluent B) containing 10 mM formic acid. The initial conditions were 100% A at a flow rate of 0.4 ml/min and were maintained for 1 min, followed by a linear gradient to 1% A at 15 min. These conditions were kept constant for 2 min and then changed to the initial conditions. The runtime was 20 min, and the injection volume was 1 µl. Samples were kept cool at 6°C in the Waters Sample Manager during the analysis.

#### 2.5.3 Time-of-flight mass spectrometer analysis

The SYNAPT G1 mass spectrometer was used in V-optics and operated in electrospray mode to enable detection of all ESI-compatible compounds. Leucine enkephalin (50 pg/ml) was used as a reference calibrant (Lock Mass) to obtain typical mass accuracies between 1 and 5 mDalton (mDa). The mass spectrometer was operated in both ESI positive and negative modes with a capillary voltage of 2.5 kV, the sampling cone at 30 V, and the extraction cone at 4.0 V. The scan time was 0.1 s covering the 50–1,200 Da mass range with an interscan time of 0.02 s. The source temperature was 120°C and the desolvation temperature was set at 450°C. Nitrogen gas was used as the nebulization gas at a flow rate of 550 L/h and cone gas was added at 50 L/h. Argon was used as collision gas during fragmentation experiments. The software used to control the hyphenated system and do all data manipulation was MassLynx 4.1 (SCN 872). Compound identification was further enhanced by analysing all samples with low and high collision energy settings of the collision cell. To minimize compound fragmentation a low energy setting of 3 V was used, but to enhance fragmentation of molecules, five different collision energy profiles between 10 and 50 V were used (MS^e^).

### 2.6 Statistical analyses

Experiments were done in triplicate with two independent assay repeats, and results expressed as mean ± standard deviation (SD). The LC_50_ values, corresponding to the concentration required to inhibit 50% of cell viability, were calculated from a sigmoidal dose-response of a non-linear regression and R-square values representing the best fit of the model were assessed using One-way analysis of variance (ANOVA) as well as to determine the differences in means, and statistical processing of the data was performed using GraphPad Prism software (Version 8.0). Tukey’s multiple comparison test was used to determine significant differences between the means of treated and untreated groups.

## 3 Results

### 3.1 Cytotoxicity results

The MTT assay was used to assess the cytotoxicity of the extracts on the African green monkey kidney Vero cell line. Extract/Acyclovir concentration ranging from 8.0 to 1,000 μg/ml were used to treat cells. The findings revealed dose-dependent toxicity, with higher cell viability at lower concentrations and a gradual decline in cell viability as concentrations increased. Table 2 lists the lethal concentrations that lowered viability of cells by 50% (LC_50_). These concentrations lower than the LC_50_ (10 μg/ml) were further tested for antiviral activity. All plant extracts exhibited a varying degree of toxicity on Vero cells with LC_50_ values greater than 20 μg/ml for most samples. These values were determined to ensure that the LC_50_ values used in this study are safe when compared to the LC_50_ value for acyclovir (positive control).

**TABLE 2:** Cytotoxic effects of leaves and stem extracts of *H. aureonitens* at different sites at two diverse geographical locations and two seasons of the year.

Collection Location	Site	Plant parts	Collection Season	Cytotoxicity LC_50_ (µg/ml)	Selectivity Index (SI)
Telperion	Site 1	leaves	Spring	17.8 ± 1.48	0.200
Telperion	Site 1	Stem	Spring	14.4 ± 0.56	0.917
Telperion	Site 2	leaves	Spring	16.0 ± 0.51	0.320
Telperion	Site 2	Stem	Spring	29.5 ± 0.25	1.050
Wakefield	Site 1	leaves	Spring	19.9 ± 2.73	0.215
Wakefield	Site 1	Stem	Spring	42.5 ± 4.92	0.662
Wakefield	Site 2	leaves	Spring	26.4 ± 4.37	0.301
Wakefield	Site 2	Stem	Spring	23.4 ± 4.89	0.835
Wakefield	Site 3	leaves	Spring	27.6 ± 0.51	0.554
Wakefield	Site 3	Stem	Spring	32.2 ± 0.44	0.994
Wakefield	Site 4	leaves	Spring	35.7 ± 1.21	0.472
Wakefield	Site 4	Stem	Spring	46.9 ± 0.43	0.640
Telperion	Site 1	leaves	Autumn	24.3 ± 6.29	1.538
Telperion	Site 1	Stem	Autumn	18.5 ± 0.45	1.170
Telperion	Site 2	leaves	Autumn	24.8 ± 1.30	0.001
Telperion	Site 2	Stem	Autumn	21.3 ± 0.21	0.687
Telperion	Site 3	leaves	Autumn	35.2 ± 0.94	0.488
Telperion	Site 3	Stem	Autumn	31.4 ± 3.43	1.468
Wakefield	Site 1	leaves	Autumn	24.2 ± 9.41	0.618
Wakefield	Site 1	Stem	Autumn	40.7 ± 3.02	0.806
Wakefield	Site 2	leaves	Autumn	24.9 ± 2.68	0.294
Wakefield	Site 2	Stem	Autumn	43.6 ± 5.68	0.509
Wakefield	Site 3	leaves	Autumn	25.6 ± 3.12	0.317
Wakefield	Site 3	Stem	Autumn	25.9 ± 3.43	0.302
Wakefield	Site 4	leaves	Autumn	27.9 ± 10.56	1.570
Wakefield	Site 4	Stem	Autumn	20.4 ± 8.39	1.284

LC_50_ figure represents extract concentration that are lethal to 50% of Vero cells. Positive control, acyclovir LC_50_ = 10 µg/ml. Selectivity Index (SI) = LC_50_ (µg/ml)/TCID_50._ The higher the SI value, the safer the extracts are.

### 3.2 Anti-HSV

**FIGURE 1:**
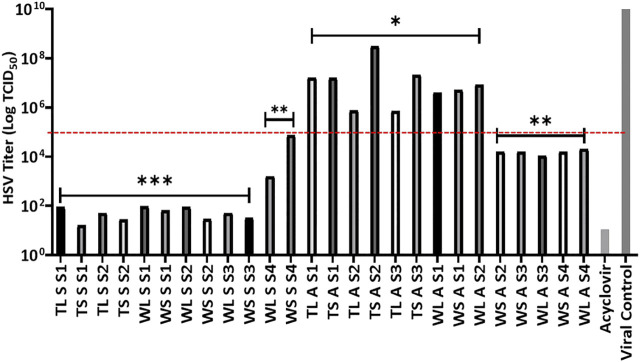
Effects of *H. aureonitens* extracts (leaves and stems) from different sites at two different locations and at two different seasons against HSV tissue culture infections dose (TCID_50_) in Vero cell culture. Mean HSV-1 titers (±SD) determined using one-way ANOVA where *p < 0.05, **p < 0.001, ***p < 0.0001. TL, Telperion leaves; TS, Telperion stems; WL, Wakefield leaves; WS, Wakefield stems; S, spring; A, autumn; S1–S4, collection site.

Plant extract concentration of 10 μg/ml was used to determine the antiviral activity, based on the non-toxic nature of the extracts as shown by the LC_50_ values against Vero cells. Further to this was the simultaneous inoculation of the combination of extract-virus inoculum to the cells in their individual wells. The tested concentration was observed to significantly decrease the viral titre as observed by the inhibition of the CPE in Vero cells caused by viral infection. Because the extracts reduce viral load by 2 logs when compared to the virus control, the HSV titre log benchmark was therefore set at 10^5^ TCID_50_ to classify activity where all extracts that reduce the viral burden to a figure below log 10^5^ were considered very active as seen in Figure 1.

### 3.3 Chlorogenic acids profile

Chlorogenic acids (CGA) are a type of natural substance that can be found in a variety of plant species, and as such are of special interest in many research studies. Since the main CGA represented in nature is caffeoylquinic acid (CQA), it is therefore often used as a quality control indicator for a variety of natural products (Gil and Wianowska, 2017). On that account, and on the presence of these compounds in *Helichrysum* species as reported in previous studies (Gradinaru et al., 2014; Grinev et al., 2016), this study hence focused on evaluating specifically CQA distribution and its derivatives of other classes of compounds namely dicaffeoylquinic acids (DCQA) and tricaffeoylquinic acids (TCQA) in the chemical profile of *H. aureonitens*. Given that a variety of mechanisms may be involved in plant metabolite production, this study investigated whether CQA accumulation in *H. aureonitens* is affected by seasonal fluctuations and the species’ growing site. The extracts were treated to ultra-performance liquid chromatography-quadrupole time-of-flight mass spectroscopy (UPLC-qTOF-MS) and Table 3 lists the compounds from the extracts that have been detected. Eleven isomers of CGA from three derivatives of CQA were detected in this study, four isomers of CQA (monocaffeoylquinic acids), six isomers of DCQA and one isomer of TCQA.

**TABLE 3:** Chlorogenic acids composition of *H. aureonitens*.

Classification	Number	Retention	Compounds	Calculated	Calculated	Detected	Mass	DBE count	MS/MS
		Time (min)		Empirical formula	Mass	Mass	Accuracy (mDa)		Fragmentation ions
CQA	1	2.79	5-CQA (Trans)	C_16_H_18_O_9_	354.0951	353.0860	1.3	8	353.1; 191.1
	2	3.47	3-CQA	C_16_H_18_O_9_	354.0951	353.0865	0.8	8	353.1; 191.1; 179.0; 135.0
	3	5.73	5-CQA (Cis)	C_16_H_18_O_9_	354.0951	353.0853	2.0	8	353.1; 191.1
	4	5.96	4-CQA	C_16_H_18_O_9_	354.0951	353.0859	1.4	8	353.1; 191.1; 173.0; 135.0
DCQA	1	6.15	3,4-DCQA (Trans)	C_25_H_24_O_12_	516.1268	515.1207	1.7	14	515.1; 353.1; 173.1; 335.1
	2	7.27	3,5-DCQA (Trans)	C_25_H_24_O_12_	516.1268	515.1203	1.3	14	515.1; 353.1; 191.1
	3	8.79	3,4-DCQA (Cis)	C_25_H_24_O_12_	516.1268	515.1181	1.1	14	515.1; 353.1; 173.1; 335.1
	4	8.95	3,5-DCQA (Cis)	C_25_H_24_O_12_	516.1268	515.1180	1.0	14	515.1; 353.1; 191.1
	5	9.08	4,5-DCQA (Trans)	C_25_H_24_O_12_	516.1268	515.1177	1.3	14	515.1; 353.1; 335.1; 173.1
	6	9.22	4,5-DCQA (Cis)	C_25_H_24_O_12_	516.1268	515.1200	1.0	14	515.1; 353.1; 335.1; 173.1
TCQA		10.14	TCQA 2	C_34_H_30_O_15_	678.1585	677.1520	1.3	20	677.2; 515.1; 353.1; 335.1; 173.1

Source: Clifford et al. (2003), Ramabulana et al. (2020).

### 3.4 Integration values comparison

The following figures show the comparison between integration values of isomers of CQA, DCQA, and TCQA across locations and seasons, with the *X*-axis presenting each compound of the isomers, while the *Y*-axis gives the integration values for the compounds. The integration values represent the concentration in quantity of each isomer and derivatives of chlorogenic acids in this study. They are depicted by the numerical data labels on each of the bars in the graph.

**FIGURE 2:**
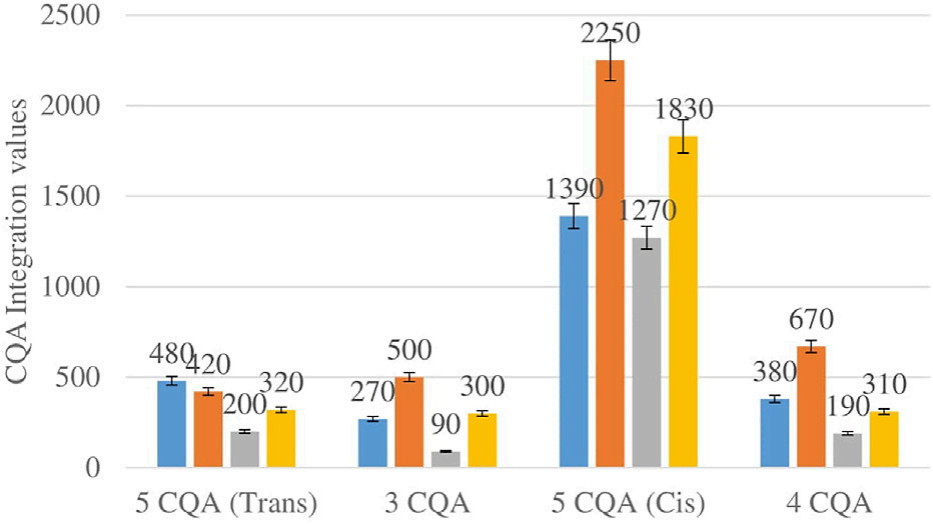
CQA isomers mean concentration comparison between wet sites in each of the two locations in both seasons. Blue-wet site spring (Telperion), Orange-wet site spring (Wakefield), Grey-wet site autumn (Telperion), Yellow-wet site autumn (Wakefield).

Figure 2 shows the comparison between the integration values of four isomers of caffeoylquinic acid (CQA 1–CQA 4) between wet sites from the two locations in both seasons. The 3-CQA, 4-CQA and 5-CQQA (cis) isomers all have higher concentrations at Wakefield compared to Telperion in both seasons.

**FIGURE 3:**
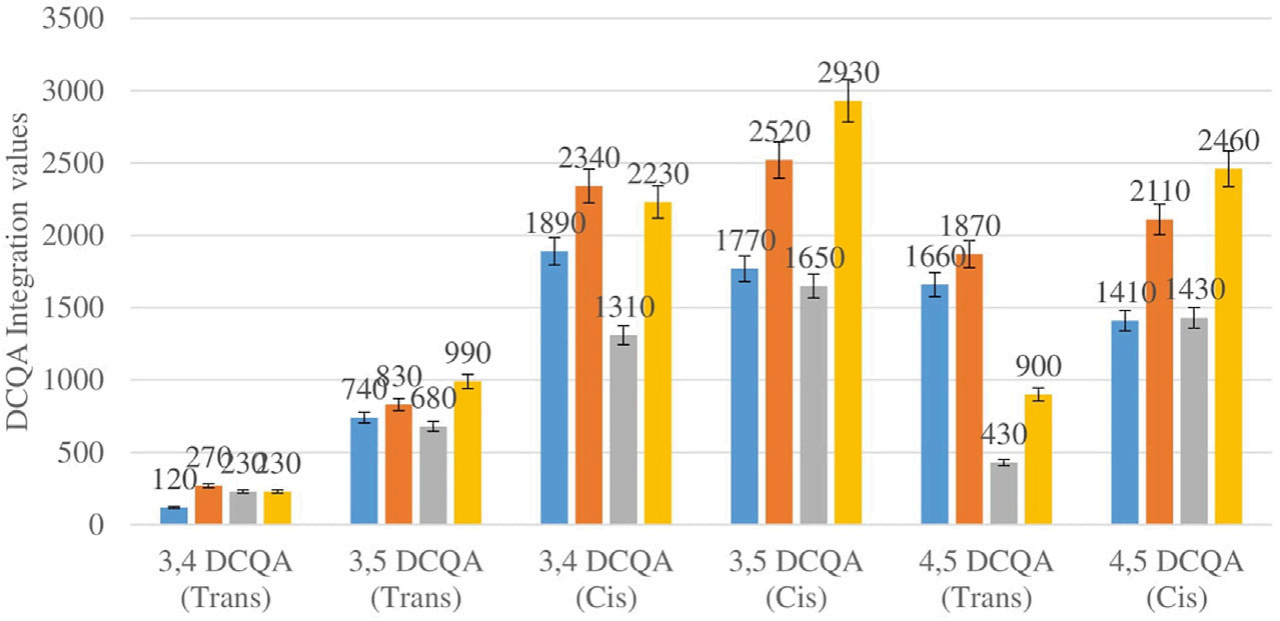
DCQA isomers mean concentration comparison between wet sites in each of the two locations in both seasons. Blue-wet site spring (Telperion), Orange-wet site spring (Wakefield), Grey-wet site autumn (Telperion), Yellow-wet site autumn (Wakefield).

Figure 3 shows the comparison between the integration values of six isomers of dicaffeoylquinic acid (DCQA 1–DCQA 6) between wet sites from the two locations in both seasons. The isomer 3,4 DCQA have the same integration value in both locations in autumn. The rest of the isomers of DCQA however were higher in concentration at Wakefield in both seasons.

**FIGURE 4:**
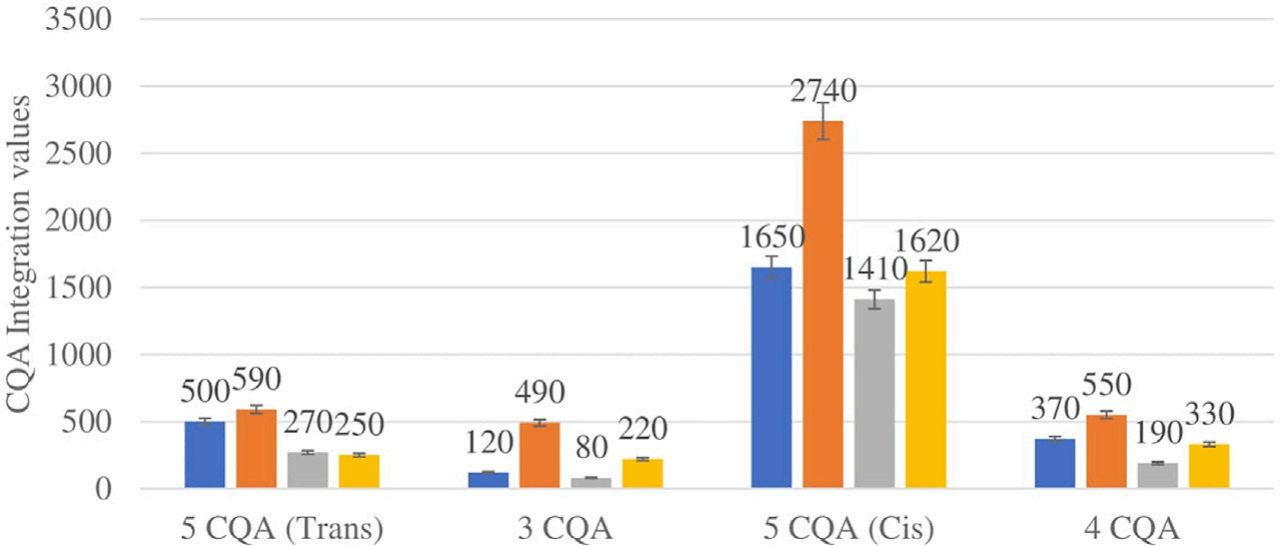
CQA isomers mean concentration comparison between wet and dry sites in each of the two locations in autumn. Blue-wet site autumn (Telperion), Orange-wet site autumn (Wakefield), Grey-dry site autumn (Telperion), Yellow-dry site autumn (Wakefield).

Figure 4 shows the comparison between the integration values of four isomers of caffeoylquinic acid (CQA 1–CQA 4) between wet and dry sites combined from each of the two locations in the autumn season. CQA concentration was higher in all the wet sites compared to dry sites.

**FIGURE 5:**
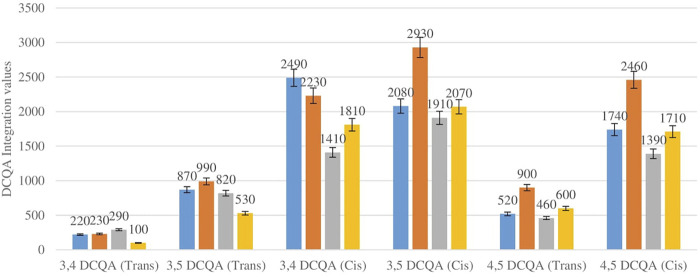
DCQA isomers mean concentration comparison between wet and dry sites in each of the two locations in autumn. Blue-wet site autumn (Telperion), Orange-Wet site autumn (Wakefield), Grey-dry site autumn (Telperion), Yellow-dry site autumn (Wakefield).

Figure 5 shows the comparison between the integration values of six isomers of dicaffeoylquinic acid (DCQA 1–DCQA 6) between wet and dry sites combined from each of the two locations in the autumn season. Higher concentrations of DCQA were also observed in the wet sites.

**FIGURE 6:**
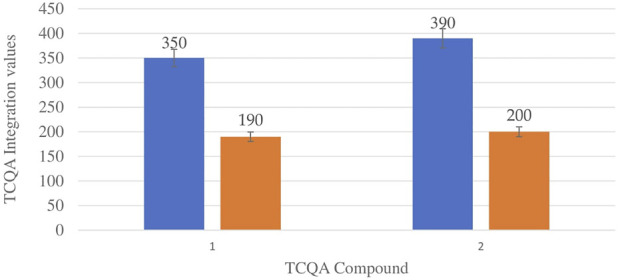
TCQA isomers mean concentration comparison between wet and dry sites from Telperion and Wakefield in autumn. 1st Blue-Wet site Telperion, 1st Orange-Dry site Telperion, 2nd Blue-Wet site Wakefield, 2nd Orange-Dry site Wakefield.

Figure 6 shows the comparison between the integration values of the only isomer of tricaffeoylquinic acid (TCQA) between wet and dry sites from both locations in the autumn season. There is a slight increase in TCQA production in Wakefield compared to Telperion while a higher concentration of the isomer was observed in the wet site compared to the dry site in both locations.

## 4 Discussion

In this study, anti-HSV activity of twenty-six extracts of *H. aureonitens* from different sites at two different locations and at two different seasons were evaluated, and the result showed varying degrees of pharmacological potency against the virus. The choice of Vero cells as the study’s primary cell line was due to its reputation as the most susceptible cell line to virus-mediated cell death and virus proliferation. They have been widely used in toxicology, virology, and pharmacology studies, as well as vaccine development and diagnostic reagent production (Wu et al., 2017; Shen et al., 2019).

The cytotoxicity result presented in Table 2 showed a general high level of toxicity of *H. aureonitens* extract across both seasons and different climatic regions with LC_50_ values ranging between 14.48 and 46.99 μg/ml. Heyman and Meyer (2012) reported a similarly high level of toxicity of the following *Helichrysum* species, *H. acutatum* (25.16 μg/ml), *H. appendiculatum* (29.01 μg/ml), *H. panduratum* (3.40 μg/ml) and *H. psilolepis* (27.07 μg/ml). *H. aureonitens* and *H. nudifolium* both have even higher cytotoxic values of <3.13 μg/ml each, very close to the positive control zearalenone with the cytotoxic value of 1.33 μg/ml. The results, although close to the current study, still have some variation with report of higher toxicity compared to the current result. This can be due to reasons such as use of different extraction solvents, variations in seasons or difference in climatic regions. Heyman and Meyer (2012) extracted with chloroform while the current study used acetone. Umar et al. (2013), in their study that compared antimicrobial properties of extracts from dried stems of *Opuntia dillenii* and rhizomes of *Zingiber officinale* using non-polar (petroleum ether and chloroform) and polar solvents (methanol and water) discovered that ether and chloroform extracts of *Opuntia dillenii* demonstrated better antibacterial efficacy against *Escherichia coli* (gram negative) when compared to methanolic and water extracts indicating that the polarity of the solvent used during extraction significantly influenced the antimicrobial activity of the plants. Eloff (1998) also compared various extractants in common use and reported different results based on individual extractants used in the isolation of antimicrobial components of plants.

The extracts at 10 μg/ml investigated in this study significantly reduced the HSV infection in Vero cells. Figure 1 shows that seventeen of the twenty-six extracts tested had significant anti-HSV activity, as evaluated by a tissue culture infectious dose (TCID_50_) reduction of less than 10^5^. The observed strong activities of the seventeen extracts as seen in the reduction in the HSV titre values is significantly better when compared to the positive control. Twelve of the seventeen were extracts from plants collected during the spring season while the remaining five were collected during autumn. Significantly higher antiviral activities of *H. aureonitens* plant extracts were recorded in the spring season in both locations, and the extracts’ activities were more than double in comparison to the activity of extracts of plants collected in autumn as shown by the significant reduction in the viral load depicted by the HSV titre values represented in Figure 1. This supports many studies that have reported the influence of seasonal variation on the antimicrobial activities of plants (Ncube et al., 2011; Ramírez-Briones et al., 2019). Further to the ongoing, it is clear that different climatic regions exert influence on the potency of *H. aureonitens* against the HSV-1. This is confirmed by a comparison between the activity of extracts from Telperion and Wakefield during the autumn season. While extracts’ activities in spring was comparatively the same (except for the dry site of Wakefield) in both locations, extracts from autumn demonstrated better activity in the dry sites at Wakefield as seen by the reduced HSV titer (Figure 1). A few studies conducted on the antiviral properties of *Helichrysum* species reported varying activities. At concentrations ranging from 12 to 47 μg/ml, galangin, isolated from *H. aureonitens*, demonstrated considerable antiviral activity against a DNA virus, HSV-1, and an RNA virus, Coxsackie B Type-1, while showing no activity against Adenovirus Type-31 (Meyer et al., 1997). Also, ethanolic extracts of *H. arenarium* and *H. armenium* showed significant antiviral activities against HSV-1 and PI-3 at concentrations of 2–32 and 4–64 μg/ml, respectively.

The *Helichrysum* genus is generally known for its variety of chlorogenic acids compounds and previous studies have highlighted the presence of CGA in many of the species (Albayrak et al., 2010; Heyman et al., 2015; Babotă et al., 2018). Chlorogenic acids, more accurately referred to as 3-CQA as per IUPAC guideline is a caffeoylquinic acid and the most abundant isomer among the other caffeoylquinic acid isomers (3-, 4-, and 5-CQA) (Naveed et al., 2018). Other derivatives of CGA accessed in this study are dicaffeoylquinic acid (DCQA) and tricaffeoylquinic acid (TCQA). Eleven caffeoylquinic acid compounds, belonging to three derivatives were identified. The first derivative which is monocaffeoylquinic acid, CQA has four isomers, the second derivative, DCQA has six isomers while the last derivative TCQA has only one isomer. Although the presence of chlorogenic acid has been confirmed in *H. aureonitens* by a number of studies, no study has however delineated the specific derivatives of chlorogenic acid that are present in the species or the most abundant of these derivatives. This study determined the various caffeoylquinic acid compounds in the three derivatives groups of chlorogenic acid in the harvested plants across two seasons (spring and autumn) and in different sites from two geographically diverse locations. LC-MS analysis was used to determine which of the caffeoylquinic acids was more abundant in response to seasonal changes. With the exception of the first isomer of monocaffeoylquinic acid, 5-CQA (Trans), the rest of the isomers of CQA all have higher concentrations comparatively at Wakefield as compared to Telperion during the spring season while all CQA isomers with no exception have larger concentrations than the Telperion sites in autumn (Figure 2). A combined comparison between the integration values of all four isomers of caffeoylquinic acid between wet and dry sites from each of the two locations in the autumn season also clearly showed a higher concentration of CQA for all wet sites at both locations, with Wakefield having relatively higher concentrations than Telperion and an observed significant increase in 5-CQA (Cis) concentration (Figure 4). Apart from an equal 3,4 DCQA integration value for both locations in the autumn season, all other DCQA integration values support higher concentration of DCQA across all the isomers in Wakefield for both seasons, with higher values recorded in spring except for 3,5 DCQA (Trans) and 4,5 DCQA (Cis) (Figure 5). With the exception of the 3,4 DCQA (Trans) at Telperion, all other values again showed a higher concentration in the wetter sites than the drier sites across the two locations. These evidently show that chlorogenic acids isomers production are increased as conspicuously seen (Figures 2–5) with increased moisture which triggers the production of both CQA and DCQA irrespective of the climatic regions. It is also observed that higher rainfall triggers the production of DCQA and CQA in wetter climatic regions than drier location (Figure 5).

TCQA levels are fairly the same in the two locations during spring. The TCQA values between wet and dry sites in both locations during autumn are comparable to each other. The wet site recorded higher concentration of TCQA (about double) above the dry site in both Telperion and Wakefield (Figure 6).

Of the three derivatives of chlorogenic acids, the most abundant is dicaffeoylquinic acid (DCQA) which has a larger concentration in both locations and in both seasons. The presence of dicaffeoylquinic acid isomers in *Helichrysum* species has already been reported by (Gouveia and Castilho, 2011) in their work on *H. obconicum* (an indigenous *Helichrysum* species from the Madeira Archipelago), where the fragmentation characterization is described extensively. In the further work of the same team (Gouveia and Castilho, 2012) their study on caffeoylquinic acids separation, quantification and identification in medicinal *Helichrysum* species (*H. devium*, *H. melaleucum*, *H. obconicum*) arrived at similar results where they reported that among the measured hydroxycinnamic acids, dicaffeoylquinic acids isomers were the most prevalent. The most abundant isomer of caffeoylquinic acid recorded in this study is 3-CQA. This is in alignment with reports from studies of other plants (Moeenfard et al., 2014; Alc Azar Maga∼Na et al., 2021).

## 5 Conclusion

The result of the study supports the outcome of many studies on the influence of seasonal variation on the antimicrobial activity of medicinal plants. It is therefore concluded that the antiviral activity of *H. aureonitens* is contingent upon a number of factors which include seasonal variation and the specific climatic region in which the plant is growing. The spring season favours a much better antiviral activity of the plant in both wet and dry locations and collections are advised to be made in spring season for effective antiviral activity. Also, given that plant metabolite synthesis can be influenced by a variety of processes, LC-MS analysis confirmed that seasonal fluctuations and the species’ growing site affect chlorogenic acid accumulation in *H. aureonitens.* The synthesis of different derivatives and isomers of each derivative in the leaves of *H. aureonitens* changes in response to seasonal variations as established with the UPLC-qTOF-MS analytical platform. Changes in season particularly played a significant role in both DCQA and CQA production in *H. aureonitens* with the highest concentration largely found in the wet season-spring. Even though there are slight differences in the concentration of compounds from both locations during autumn, the chemical profile remained largely the same. A comparison between the CQA content of *H. aureonitens* growing in two geographically diverse locations also showed that the levels of CQA is favoured in the wetter geographical location-Wakefield, Midland in KwaZulu-Natal. The study therefore concludes that chlorogenic acids isomers is increased with increased moisture levels which triggers the production of both CQA and DCQA irrespective of the climatic regions in *H. aureonitens.*

## Data Availability

The original contributions presented in the study are included in the article/supplementary material, further inquiries can be directed to the corresponding author.
